# Thermoplastic Intumescent Coatings Modified with Pentaerythritol-Occluded Carbon Nanotubes

**DOI:** 10.3390/ma14216284

**Published:** 2021-10-21

**Authors:** Michał Tomczak, Jakub Łopiński, Agnieszka Kowalczyk, Krzysztof Kowalczyk

**Affiliations:** 1 Chemical Alliance Polska, Prosta 23, 72-100 Łozienica, Poland; Michal.Tomczak91@gmail.com; 2 Department of Chemical Organic Technology and Polymeric Materials, Faculty of Chemical Technology and Engineering, West Pomeranian University of Technology in Szczecin, Piastów Ave. 42, 71-065 Szczecin, Poland; Jakub.Lopinski@zut.edu.pl (J.Ł.); Agnieszka.Kowalczyk@zut.edu.pl (A.K.)

**Keywords:** carbon nanotubes, intumescent layer, nanofiller, pentaerythritol, solid dispersion

## Abstract

A thermoplastic intumescent coating system (IC) based on poly(vinyl acetate) was modified by two forms of multiwalled carbon nanotubes (CNTs), i.e., by a nanofiller powder and its solid dispersions in pentaerythritol (PER-CNTs). It was revealed that only the PER-CNTs modifier allows us to obtain solvent-borne ICs with a relatively high CNTs concentration (1–3 wt. parts of CNTs/100 wt. parts of paint solids) and acceptable application viscosity. Thermal insulation time (TIT) and intumescent factor (*IF*) of the ICs on a steel substrate (a fire test according to a cellulosic fire curve), as well as morphology, chemical structure (by the FT-IR technique) and mechanical strength of the charred systems, were investigated. It was found that the CNTs powder decreases TIT and *IF* values while PER-occluded CNTs improve these parameters (e.g., +4.6 min and +102% vs. an unmodified sample, respectively). Compressive strength of the charred ICs was improved by the PER-CNTs modifier as well.

## 1. Introduction

For the first time, an intumescent process of organic coatings was widely described in 1971 [1]. Since then, researches have been focused on characterization, refining and modification of this phenomenon. To the basic components of conventional intumescent coatings (ICs) belong (i) an inorganic acid or a compound releasing this acid during heating (mainly ammonium polyphosphate; APP), (ii) a carbon-rich substance allowing the formation of a char (pentaerythritol; PER) and (iii) a blowing agent facilitating foaming of the entire system (melamine, MEL). The char-forming process is much more complicated than the simple chemical reactions occurring between the main components; the process is the result of simultaneously charring and foaming of the coating surface [2,3,4,5,6]. The resulting char is an insulating layer that slows the heat and mass transfer between the gas and the condensed phase. Mechanical strength of the formed chars is another important feature of the intumescent systems. Real fire conditions consist of not only high temperature gases but also their intensive movement in the fire area; it can lead to char destruction and its removal from protected steel elements.

**Figure 1:**
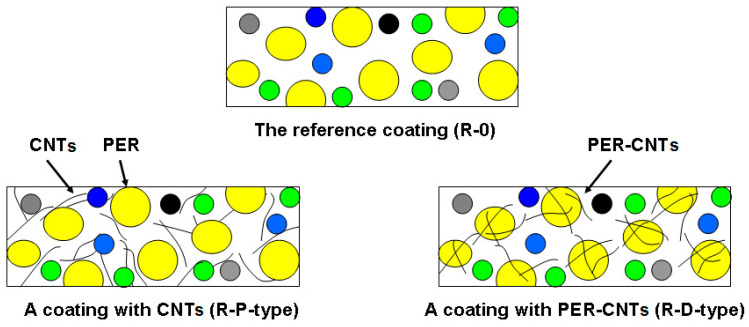
Schemes of structures of the tested intumescent coatings (i.e., the reference sample and the samples with CNTs or PER-CNTs solid dispersions).

The efficiency of thermal insulation of intumescent bulk and coating materials can be significantly improved by relatively small doses of various modifiers, especially nanofillers, e.g., boron compounds [7,8], silica and silicates [9,10,11,12], metal oxides (ZnO, MnO_2_, Ni_2_O_3_, Bi_2_O_3_, TiO_2_, ZrO_2_) [13,14,15,16] or bio-CaCO_3_ [17]. Cellulose nanocrystals and candle soot nanoparticles seem to be interesting carbon-rich components for ICs as well [18,19]. In a few cases, an incorporation of carbon nanofillers into the thermoplastic and thermosetting ICs improves their thermal and/or mechanical features; however, the effect is strongly affected by a structure of the modifiers. For example, it was revealed that graphene powder (Gn) addition positively influences the thermal insulation efficiency as well as the compressive strength of charred thermoplastic water- [20] and solvent-borne systems [9]. On the other hand, it was significantly demonstrated [21] that CNTs negatively affect all the tested features of acrylic ICs. This phenomenon was explained by a disturbance in a swelling process by tangled CNT particles. It is generally known that CNTs significantly increase the viscosity of various coating binders (e.g., [22]) while graphene-modified polymeric materials do not exhibit any visible changes in this parameter (platelet-type particles of this carbon nanofiller create irregular aggregates). To the best of authors’ knowledge, the modification of thermoplastic-type ICs by CNTs was not presented and described elsewhere (i.e., except for [21]). Considering the above-mentioned negative impact of the CNT powder on the crucial parameters of thermoplastic ICs, a new method of the nanofiller incorporation into these systems was developed. In this paper, CNTs were physically occluded/coated by PER and tested in poly(vinyl acetate)-based intumescent paints. This novel technique allows us to separate and “freeze” the CNT bundles in PER particles, and thus it reduces the negative influence on the thermoplastic binder features. This is possible to realize due to significant differences of the PER water solubility at room (72 g/L) and elevated temperature (354 g/L, 75 °C) [23]. For comparison, ICs containing powdered PER and CNTs were prepared and tested as well (Figure 1).

## 2. Materials and Methods

### 2.1. Materials

The intumescent paints and coatings were prepared using the following components:

(1) Poly(vinyl acetate) (PVAc) with the average molecular weight of 176,000 g/mol, density ca. 1.2 g/cm^3^, glass transition temperature ca. 42 °C, softening temperature 150 °C and solid content >99% (M50; Synthomer, Essex, UK);
(2) Ammonium polyphosphate (type II) (APP), a powder with the average particles diameter of 18 µm (FR Cross 484; Budenheim, Germany);
(3) Melamine (MEL), a powder with a particles size ≤40 µm (Melafine; OCI Nitrogen, Amsterdam, The Netherlands);
(4) Pentaerythritol (PER), a powder with a particles size ≤40 µm, density ca. 1.4 g/cm^3^ (Dispersion&Resins, Włocławek, Poland);
(5) Multi-walled carbon nanotubes (CNTs), a powder with a particle length ≤1.5 µm (Nanocyl NC7000; Nanocyl, Sambreville, Belgium);
(6) An aqueous dispersion of Nanocyl NC7000 (3 wt.% of CNTs) (Aquacyl AQ0302; Nanocyl, Sambreville, Belgium);
(7) Titanium dioxide (a rutile type) with the average particles size of 290 nm (Tytanpol R-001; GA Z.Ch. Police, Poland);
(8) Zinc borate heptahydrate (ZB) with the particles size ca. 40 µm (POCh, Gliwice, Poland);
(9) A wetting/dispersing additive based on hydroxyl-functional carboxylic acid ester (Disperbyk 108; BYK-Chemie, Wesel, Germany);
(10) A silicone defoamer (Byk-066N; BYK-Chemie);
(11) n-Butyl acetate as a solvent (Chempur, Piekary Śląskie, Poland).

### 2.2. Sample Preparation

#### 2.2.1. Preparation of the Solid CNTs Dispersions in PER

The PER powder was dissolved (under vigorous mixing) in water containing an appropriate amount of the aqueous CNT dispersion heated to 80 °C. Then, the system was agitated at this temperature for 1 h and cooled to RT (under vigorous mixing). Water was evaporated from the precipitated PER-CNT solid dispersion at 40 °C in a vacuum heater and the product was milled and sieved using an air jet sieving machine (45 μm; LPzB-2e, Multiserw-Morek, Brzeźnica, Poland). For comparison, the PER powder (without the CNTs addition) was dissolved in hot water, cooled, dried, milled and sieved as well. The CNT content in the PER-CNTs solid dispersions (three types) refered to the CNT dose of 1 wt. part (PER-CNTs-1), 2 wt. parts (PER-CNTs-2) or 3 wt. parts/100 wt. parts of paint solids (the PER-CNTs-3 solid dispersion).

#### 2.2.2. Preparation of the Intumescent Paints and Coatings

PVAc was dissolved in butyl acetate and mixed (500 rpm, 15 min) with the auxiliary additives using the laboratory dissolver with a heavy-duty dispersion impeller (VMA Getzmann GmbH, Reichshof, Germany). Then, APP, MEL, PER, as well as TiO_2_ and ZB, were added to the composition and homogenized at 6000 rpm for 30 min. The prepared reference paint was abbreviated to R-0. In other cases, the CNTs powder or its solid dispersions in PER (i.e., PER-CNTs instead of PER) was incorporated with the filler mixture. Samples modified with the CNT powder were abbreviated to R-P1 (1 wt. part) and R-P2 (2 wt. parts), while paints containing the PER-CNT solid dispersions were noted as R-D1 (1 wt. part), R-D2 (2 wt. parts) and R-D3 (3 wt. parts of CNTs/100 wt. parts of paint solids; Table 1). 

Due to the very high viscosity of the paint (R-P3) containing 3 wt. parts of CNTs (the powder form), that sample was not tested. The coating compositions (65% of solids) were applied by means of an adjustable gap applicator (Ascott, Staffordshire, UK) onto a low-carbon steel substrate with the dimensions of 100 × 100 × 0.81 mm (Q-Panels, Q-Lab Europe, Bolton, UK). The coatings were dried at RT for 5 days and then at 45 °C for 15 h. Thickness of the dry intumescent layers was 1 ± 0.05 mm. 

### 2.3. Methods

Viscosity of the solvent-borne intumescent paints was tested by means of the I.C.I. cone/plate-type viscometer (Research Equipment Ltd., London, UK). Thickness of the dry intumescent coatings was measured with the electronic film gauge (Byko-test 8500; BYK-Gardner, Geretsried, Germany) according to PN-EN ISO 2808. The thickness after a fire protection test was determined by means of a dial gauge with a stand (five measurements for each sample). Thermal analysis of the intumescent coatings was carried out using the differential scanning calorimeter (20–400 °C, a heating rate of 50 °C/min; DSC Q100, TA Instruments, New Castle, DE, USA) and the thermogravimetric analyser (50–900 °C, a heating rate of 50 °C/min, air atmosphere; TGA Q5000, TA Instr.).

A gas flame-heated programmable laboratory furnace (with two sockets for steel plates covered with the tested coating) was used for the measurement of thermal insulation features of the prepared intumescent coatings [24]. Temperature in the furnace chamber was automatically regulated (acc. to the standard cellulosic fire curve described in PN-EN 1991-1-2:2006) by a control unit of a gas burner fed with a propane–butane mixture. That temperature value as well as the temperature of the steel substrates were monitored using K-type thermocouples and a PC unit. Thermal insulation time (TIT) was presented as a time needed to reach the temperature of 450 °C (the critical temperature value) measured on a backside of the tested sample.

SEM images of the PER-CNTs-3 solid dispersion were recorded using the SU-70 microscope (Hitachi, Tokyo, Japan) while digital microscopic images of the charred layer fractures (after the furnace test) were prepared by means of the laser scanning microscope (VK-9700; Keyence, Osaka, Japan). Intumescent factor values (*IF*) for the charred intumescent coatings were calculated according to Equation (1):

(1) $$IF={\frac{L_{1}-L}{L_{o}}}_{}\left(a.u.\right)$$

where: *L* is the thickness of a steel substrate (i.e., 0.81 mm), *L*_0_ is a thickness of a dry coating before the furnace test and *L*_1_ is a thickness of the sample after the test.

Compressive strength of the charred intumescent coatings (i.e., compressive stress at the strain of 5, 10, 15, and 20% at RT) was monitored using the Z010 machine (Zwick/Roell, Ulm, Germany) equipped with a steel flat indenter with a diameter of 20 mm (indentation speed of 10 mm/min). Selected chars were also investigated using Fourier Transform Infrared Spectroscopy (FTIR) with attenuated total reflectance (ATR) accessories (Nexus FT-IR; Thermo Nicolet, Waltham, MA, USA).

## 3. Results

### 3.1. Furnace Test Results and Intumescent Coatings Features

**Figure 2:**
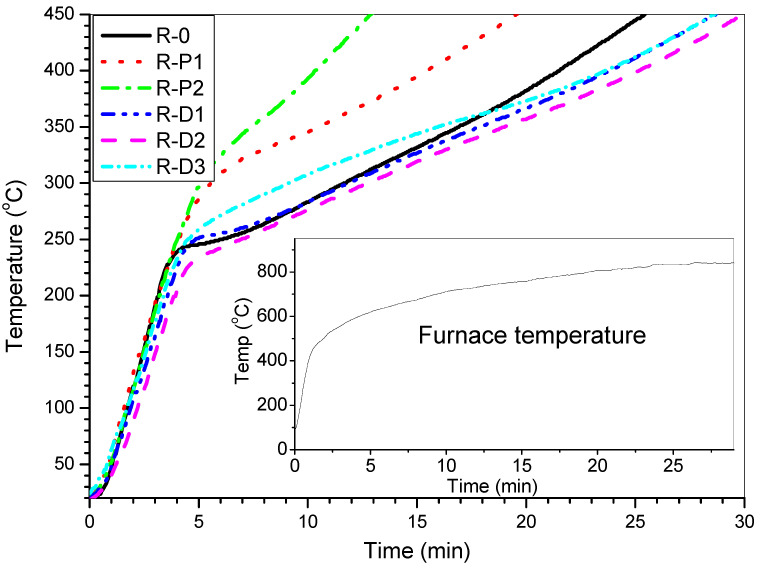
Thermal insulation curves for intumescent coatings with CNTs or PER-CNTs solid dispersions (measured during a furnace test).

Thermal insulation curves (registered during the furnace tests) for the reference coating (R-0) as well as for coatings modified with the CNTs powder (R-P-type systems) or powdered PER-CNTs solid dispersion (R-D-type systems) are shown in Figure 2; additionally, values of thermal insulation time (TIT) for these materials are presented in Table 2.

**Figure 3:**
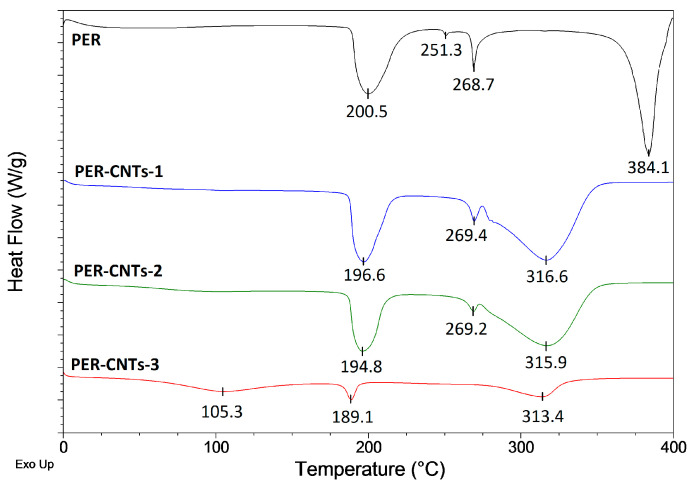
DSC curves for PER and PER-CNTs solid dispersions.

**Figure 4:**
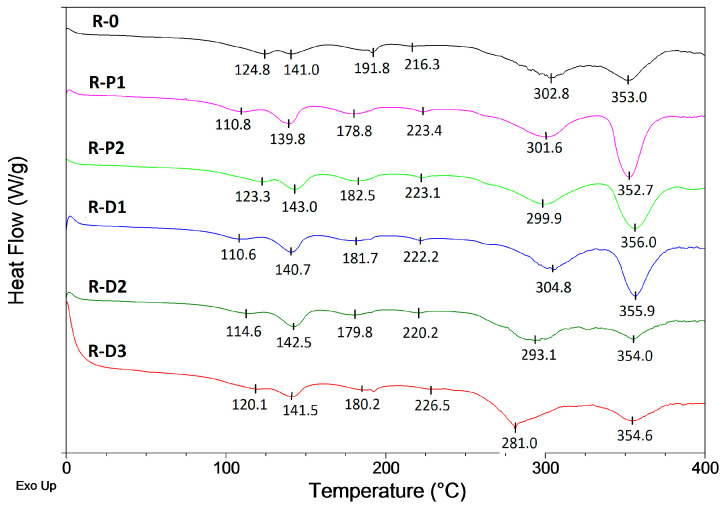
DSC curves for intumescent coatings with CNTs or PER-CNTs solid dispersions.

As can be seen, all the coatings filled with the PER-CNT solid dispersions reached significantly higher values of the TIT parameter in relation to R-0 and the other systems. In detail, the longest protection time was recorded for the sample containing 2 wt. parts of CNTs occluded by PER (R-D2, ca. 30.0 min; +4.6 min vs. R-0) while the shortest TIT was noted for the coating with 2 wt. parts of the CNTs powder (R-P2, 12.9 min; −12.5 min vs. R-0). Considering the shape of the thermal insulation curves registered for the reference (TIT = 25.4 min) and the modified coatings, many significant differences can be noted. At the beginning of the test (furnace temperature lower than 550 °C; 0–4 min; Figure 2), the temperature of the steel substrate covered with the R-D-type samples (especially with R-D1 or R-D2) was lower in relation to the other samples (however, temperature values for R-P1, R-P2, and R-0 samples were quite similar). Arguably, the char forming phenomenon was affected by (i) thermal processes occurring in the PER-CNTs solid dispersions (during fire heating of the coating systems) and surprisingly by (ii) supposed relatively high thermal conductivity of the PER-CNTs particles. DSC analyses of the PER and PER-CNTs solid dispersions (Figure 3) revealed that endothermic processes (corresponding to allotropic transition of PER from a tetragonal form to a cubic lattice structure [9] as well as melting and boiling processes) were significantly disturbed by the carbon nanofiller. However, CNTs reduced the total enthalpy value of these processes in the temperature range of 50–400 °C (from ca. 1176 J/g for the pure PER to only 544 J per 1 g of PER in the PER-CNTs-3 dispersion-probably by its crystalline structure disturbance by the CNTs particles), they occur at a markedly lower temperature in relation to the unmodified PER. Thus, during the furnace test, PER-CNTs particles (dissipated in the coatings) effectively absorb heat at a lower temperature in relation to the PER particles located in the R-0 sample. That phenomenon was observed in DSC thermographs for the intumescent coatings filled with these fillers (mainly for R-D3; Figure 4); the thermal processes revealed by the DSC technique for the reference IC have been deeply described in [9]. On the other hand, a high CNT concentration should increase the thermal conductivity of the modified PER. In the case of relatively low distances between the PER-CNTs particles, heat transfer from a coating surface to the steel substrate should be mainly realized via the PER-CNTs-based structure (during the furnace test, the PER-CNTs particles should be heated before other coating components). 

**Figure 5:**
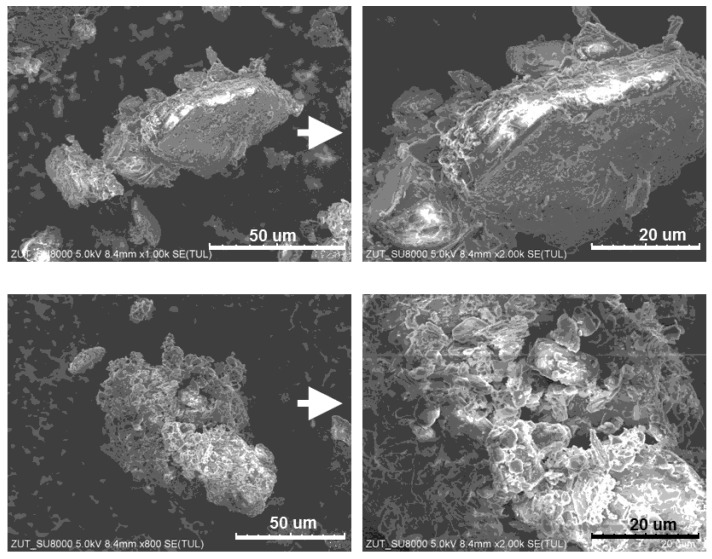
SEM images of the PER-CNTs-3 particles.

This means that the high concentration of CNTs reduces the abovementioned positive influence of this carbon nanofiller presence (in the PER-CNTs particles), i.e., the shift of the allotropic transition, melting and boiling temperatures to lower values. Thus, the steel substrate covered by the coating with the highest CNT content (R-D3) exhibited a higher steel temperature at the beginning of the furnace test (the furnace temperature lower than 550 °C; 0–4 min; Figure 2) than R-D1 and R-D2, and the temperature values for R-D3 and R-0 were quite similar. Probably, the coatings modified with the CNT powder (R-P1, R-P2) do not exhibit a higher thermal conductivity than the relevant samples with PER-CNTs particles. Generally, the solid dispersions consist of CNTs located inside and/or on the surface of the PER particles. SEM images of PER-CNTs-3 (i.e., the powder with the highest CNTs concentration) showed no CNT bundles outside the grains; the powder contains mono- and polycrystalline-type particles (Figure 5). On the other hand, the R-P-type coatings contain the carbon nanofiller particles/bundles dissipated in the polymeric matrix (PVAc) (Figure 1). Considering a lower volumetric content of the PER particles in the coating systems (13 wt. parts = 9.3 vol. parts) in relation to the PVAc matrix (14.4 wt. parts = 12 vol. parts; Table 1), it could be claimed that CNT concentration (and thermal conductivity) is higher in the PER-CNTs-based phase (the R-D-type coatings) than in the PVAc phase (the relative R-P-type samples) (Figure 1). Therefore, the R-P1 and R-P2 samples did not reach a higher steel substrate temperature in relation to R-0 and R-D-type systems (the furnace temperature <550 °C; 0–4 min; Figure 2).

**Figure 6:**
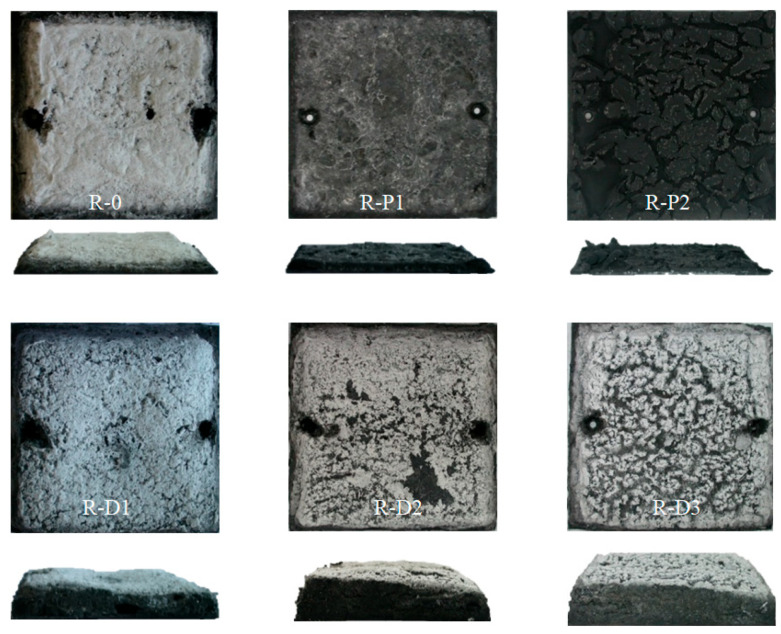
Digital images of intumescent coatings with CNTs or PER-CNTs after the furnace test.

Above the aforementioned temperature of the furnace (i.e., >550 °C), thermal insulation for the R-P1, R-P2, as well as for the R-D3 coating (the highest CNTs concentration in the PER-CNTs particles), were significantly reduced in relation to the unmodified system. Nevertheless, different mechanisms influenced this phenomenon. In the case of the R-P-type systems, CNTs were dissipated in the continuous phase of the coatings (i.e., in the PVAc binder). It is generally known that many nanofillers increase the viscosity of polymers (thus R-P3 sample was not prepared due to extremely high viscosity of the paint). Perhaps, a relatively high viscosity of the melted R-P1 and R-P2 coatings (viscosity values for the paints are presented in Table 2) disturbed a foaming process, which is represented by a “knee” of the thermal curve for R-0 (ca. 240 °C, 3–5 min; Figure 2). Above this temperature, the heating rate of the R-0 substrate was significantly reduced by the creation of a foamed char structure. In the case of the CNT powder-based samples, a slight curve inflection was observed at ca. 300 °C (R-P1) and 315 °C (R-P2). Finally, the TIT parameter was limited from 25.4 min (R-0) to 19.6 min (R-P1) and 12.9 min (R-P2; Table 2). The abovementioned limitation of the foaming process by CNTs is confirmed by intumescent factor values for these samples; the *IF* parameter was reduced from 9.9 a.u. (R-0) to 4.4 a.u. (R-P1) and 1.3 a.u. (R-P2; Table 2, Figure 6). Similar results, i.e., a reduction in the *IF* of acrylic intumescent coatings after a direct incorporation of MWCNTs were presented in [21]; however, CNT content in the systems was relatively low (only 0.5 wt.%). In this case, it is noteworthy that a powder of graphene does not reduce *IF* of the PVAc-based ICs and increses their TIT value from 25.4 to 28.7 min [9]. Nevertheless, the tested PER-CNT solid dispersions are a more effective modifier of the latter parameter.

On the other hand, a foaming process of the R-D3 coating was not disturbed by CNTs located in the PER-CNTs-3 solid dispersion, but the observed increment of the steel substrate temperature (vs. R-0; Figure 2) was resulted by the high thermal conductivity of the solid dispersion. This sample contained the highest amount of CNTs (in relation to all the tested systems); however, CNTs were located in the PER particles (with the lower volumetric concentration in relation to PVAc). Perhaps, only in this material (PER-CNTs-3), a CNT percolation threshold was achieved, because shapes of the thermal curves were quite similar only for R-0, R-D1, and R-D2 coatings (>550 °C). Nevertheless, finally, the TIT values for all the R-D-type samples were markedly higher in comparison with R-0. Interestingly, this parameter values were almost the same for the PER-CNTs-based systems containing the lowest (R-D1; 28.7 min) or the highest doses of CNTs (R-D3; 28.8 min). On the other hand, the longest insulation time was noted for R-D2 (30.0 min), but this system reached a medium *IF* value (17.9 a.u.) in relation to R-D1 (17.6 a.u.) and R-D3 (20.0 a.u.; Table 2). This shows that CNT content in the PER-CNTs solid dispersions positively affects the foaming process (a linear dependence; +10.1 a.u. for R-D3 vs. R-0) and thermal insulation features of the coatings (non-linear association; +4.6 min for R-D2 vs. R-0). These differences are caused by two main factors: a heating rate of the whole coating layer, as well as heat transfer by the layer (from fire to steel substrate). It was described that foaming processes of a typical intumescent coating progress gradually [12,25]; thus it seems that fast heating of a whole (highly thermally conductive) layer may initiate an efficient foaming process in a shorter period. In this case, a foamed char layer is formed faster and this process is directly affected by the CNT concentration. Nevertheless, thermal conductivity of the foamed char increases with the increasing CNT content in the system. Perhaps, a percolation threshold of CNTs was only achieved in the initial R-D3 sample and lasted for 18 min of the furnace test. As can be seen in Figure 2, the heating rate of the steel substrate with R-D3 coating was reduced after initiation of the foaming and carbonization processes at the furnace temperature of ca. 550 °C (4 min of heating). Probably, the CNT particles were continuously dissipated from PER-CNTs-3 particles in the whole char layer (during its foaming process). Finally (i.e., after 18 min of heating; furnace temp. of ca. 770 °C), the formed R-D3 char was already characterized by a lower thermal conductivity than the R-0 char (due to the higher *IF* value noted for R-D3).

**Figure 7:**
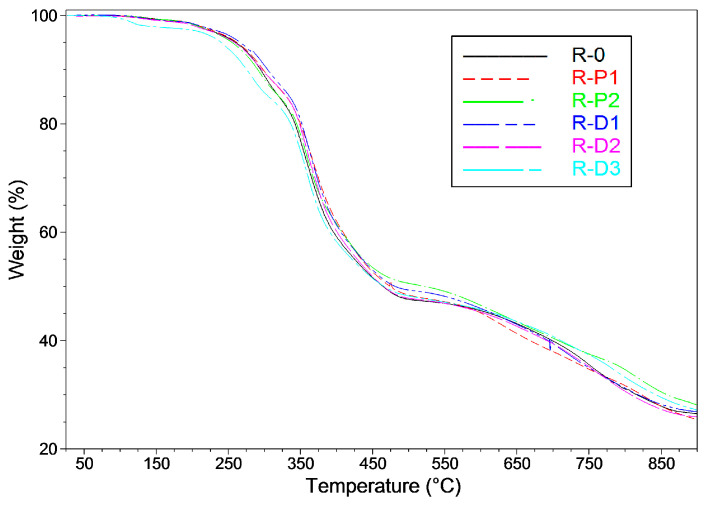
Thermogravimetric curves for intumescent coatings with CNTs or PER-CNTs solid dispersions.

Interestingly, the TITs values for the PER-CNTs-based samples correlate with thermogravimetric test results for these systems (Figure 7, Table 2). As can be seen, the calcination residue for R-D2 was slightly smaller (25.9 wt.%; TIT = 30.0 min) in relation to R-D1 (26.9 wt.%, 28.7 min) and R-D3 (27.1 wt.%, 28.8 min). Perhaps a lower viscosity value of the melted R-D2 in relation to R-D3 (although the unmelted PER-CNTs particles do not affect viscosity of the R-D-type paints; Table 2) and a faster process of the intumescent layer creation (due to its higher thermal conductivity than R-D1) were resulted in a higher volume of gaseous products released from the sample during its pyrolysis. Thus, R-D2 exhibited slightly lower calcination residue in comparison with the other PER-CNTs-based samples. On the other side, a relatively highest viscosity of the melted R-D3 (in relation to R-0 and the other R-D-type samples) caused limited evaporation of the pyrolysis products—in this case, the highest calcination residue as well as the *IF* value were noted. It seems that these features (and TIT) are mainly affected by viscosity and thermal conductivity of the melted intumescent coatings (the phenomenon was resulted by the CNTs addition). 

### 3.2. Features of Charred Intumescent Coatings

The digital images of the charred intumescent coatings are presented in Figure 6. Despite the differences of the *IF* values for the reference and modified samples, a lack of a bright sludge on the surface of the coatings filled with the CNTs powder (R-P1 and R-P2). Generally, the white surface indicates the presence of TiP_2_O_7_ obtained from a reaction between TiO_2_ and APP [26]. Considering the fact that R-D-type samples were covered by the mentioned white product, its creation was probably rather physically disturbed by the carbon nanofiller. Arguably, a too high viscosity of melted coatings with the CNTs powder negatively affected the foaming process of the charred layer, but it also reduced physical contact of the TiO_2_ and APP components (or their derivatives producing TiP_2_O_7_). It can be observed that *IF* and whiteness of the R-P-type charred samples decrease with increasing content of the powdered CNTs in the materials.

**Figure 8:**
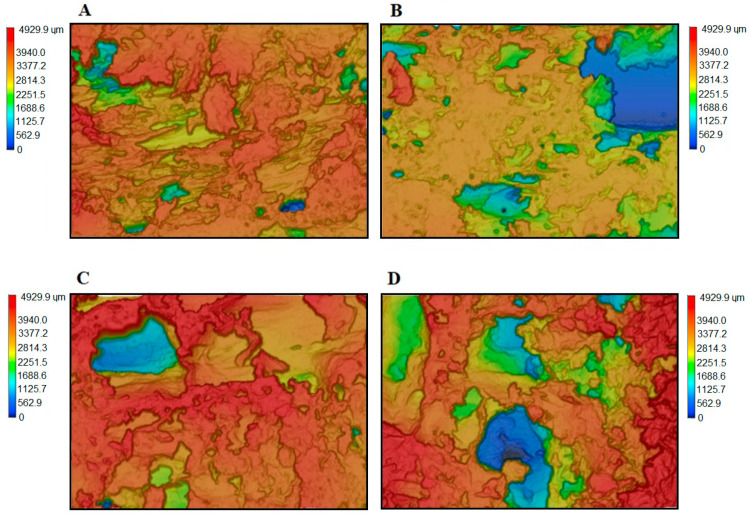
Microscopic images of the charred intumescent coatings with PER-CNTs solid dispersions: R-0 (**A**), R-D1 (**B**), R-D2 (**C**), and R-D3 (**D**).

**Figure 9:**
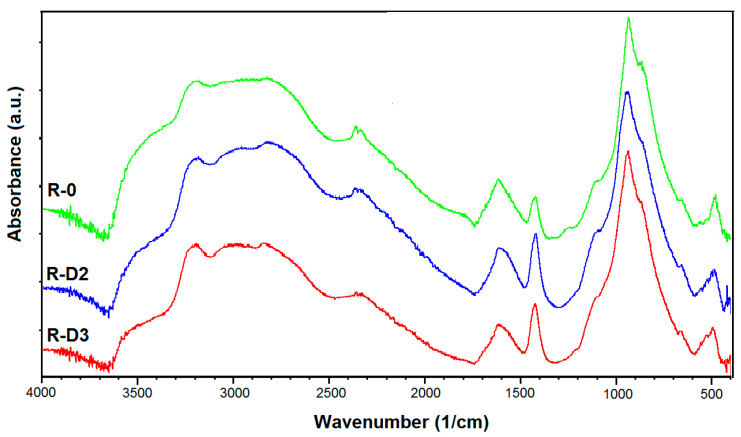
FTIR spectra for selected charred coatings with PER-CNTs solid dispersions.

In the case of the samples based on the PER-CNTs solid dispersions, the *IF* value increases with increasing content of the carbon nanofiller (while whiteness intensities of their surfaces were similar). Microstructures of these charred materials are presented in Figure 8. As can be seen, the R-D-type chars contain more and significantly larger pores in relation to the reference material. However, it is generally known that larger cells of a charred sample positively influences its thermal insulation features (TIT) [27] and this relationship does not consider the presence of a thermal conductive nanofiller in the analyzed structure. Thus, the system with the highest *IF* value (R-D3; 20.0 a.u.) did not reach the highest TIT value in relation to the other materials with the PER-CNTs solid dispersions. Nevertheless, the facts that (i) *IF* and number/size of pores located in the R-D charred coatings directly depend on the CNTs content (Table 2, Figure 6 and Figure 8), and (ii) FTIR spectra for these materials and the reference sample are deeply similar (Figure 9), may confirm that the carbon nanofiller only physically affects the features of the PER-CNTs-based systems. It is noteworthy that incorporation of powdered graphene into PVAc-based intumescent coatings significantly affected its chemical structure in relation to an unmodified system [9].

**Figure 10:**
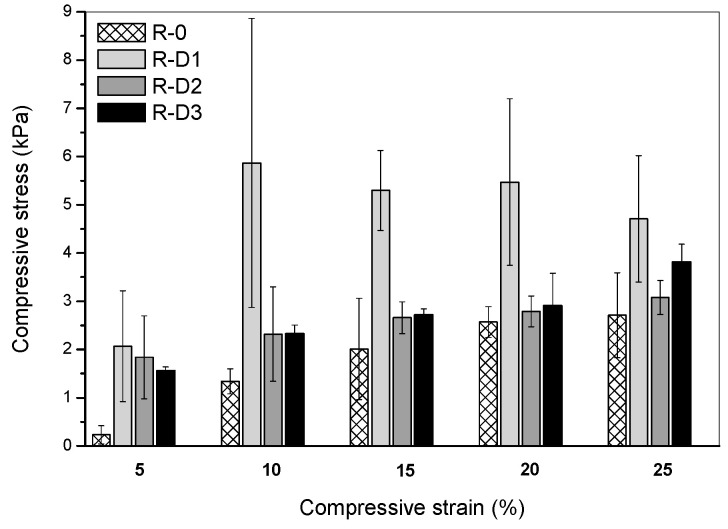
Compressive stress (at the selected strain) for charred intumescent coatings with PER-CNTs solid dispersions.

Mechanical endurance of the chars arising during rapid heating (e.g., during a fire) is a crucial parameter in the assessment of fireproof properties. During the fire, intensive movement of hot air masses occurs and it may damage/remove the charred coating; thus, its mechanical resistance is essential for an effective thermal protection of a steel substrate. Results of the compression test, i.e., compressive stress at the strain of 5, 10, 15, 20 and 25% of the investigated systems with PER-CNTs, are presented in Figure 10. Due to the low TIT and *IF* values, the R-P-type systems were not investigated. As can be seen, the carbon nanofiller addition improves the compression resistance of the charred systems and, interestingly, the largest increment of this parameter value was noted for the R-D1 material. Generally, it can be claimed that the compressive stress value (needed to reach the selected compressive strain of the PER-CNTs-based systems) decreases with increasing CNTs content and *IF* values. Arguably, that interaction was resulted from cracks observed on the surfaces of R-D-type chars (Figure 6). The surfaces of the samples exhibiting the higher *IF* values (especially R-D3) were not as smooth as the less foamed system (R-D1). These discontinuities could initiate a damage process of the charred structure during its compression. On the other hand, the smoothest charred sample (R-0) exhibited a lower compression resistance (at the lower strain values) than R-D1 and the other materials with the PER-CNTs solid dispersions. Probably, the foamed structures of the latter systems consist of large pores with cell walls perpendicularly oriented to the steel substrate and the char surface. The specific structure of the R-D-type samples may improve the compression resistance of these material (vs. the reference sample with lower *IF* value). It is noteworthy that CNT presence in the charred samples may also affect their mechanical features, but this was not proven because the higher CNT concentration in the samples did not increase the compression resistance of the modified samples. 

## 4. Conclusions

The aim of the research was to investigate the influence of PER-occluded multi-walled carbon nanotubes (the PER-CNTs solid dispersions) on thermal, chemical and mechanical properties, as well as the morphology of thermoplastic intumescent coatings based on poly(vinyl acetate). For comparison, CNTs in a powder form were tested as well. The main conclusions are as follows:

(1) Utilization of CNTs in the form of their solid dispersion in PER (PER-CNTs) allows to obtain intumescent solvent-based paints with an acceptable application viscosity while the CNT powder incorporation causes a significant increment of this feature as well as negatively influences the main properties of the dry coatings (i.e., thermal insulation time (TIT) and intumescent factor (*IF*)).
(2) The coatings filled with PER-CNTs reached markedly larger TIT and *IF* values than a reference sample (R-0); the best thermal insulation features (+4.6 min vs. R-0) were observed for the system containing 2 wt. parts of CNTs occluded by PER (per 100 wt. parts of coating components). Slightly lower TIT values were recorded for the PER-CNTs-based samples containing 1 or 3 wt. parts of CNTs (ca. +3.3 min vs. R-0). The *IF* parameter increased with the increasing CNT content (+10.1 a.u. at 3 wt. parts of CNTs).
(3) The PER-CNTs-based coatings created chars (during a fire test) with markedly better mechanical properties in relation to the unmodified sample; however, the highest compression strength was recorded for the system with the lowest CNT concentration.
(4) CNTs in the form of the PER-CNTs solid dispersions do not affect chemical composition of the charred coatings (no differences for FTIR spectra of the reference and modified samples were observed).

## Figures and Tables

**Table 1:** Intumescent coating composition.

Coating Symbol/Component (wt. Part)	R-0	R-P1	R-P2	R-D1	R-D2	R-D3
PVAc	14.4	14.4	14.4	14.4	14.4	14.4
APP	39.0	39.0	39.0	39.0	39.0	39.0
MEL	19.5	19.5	19.5	19.5	19.5	19.5
PER	13.0	13.0	13.0	0	0	0
CNTs	0	1.0 ^1^	2.0 ^2^	0	0	0
PER-CNTs	0	0	0	14.0 ^3^	15.0 ^4^	16.0 ^5^
TiO_2_	5.0	5.0	5.0	5.0	5.0	5.0
ZB	5.0	5.0	5.0	5.0	5.0	5.0
Additives	4.1	4.1	4.1	4.1	4.1	4.1

^1^—the addition of 1 wt. part of CNTs/100 wt. parts of paint solids; ^2^—the addition of 2 wt. parts of CNTs/100 wt. parts of paint solids; ^3^—PER-CNTs-1 (1 wt. part of CNTs/100 wt. parts of paint solids); ^4^—PER-CNTs-2 (2 wt. parts of CNTs/100 wt. parts of paint solids); ^5^—PER-CNTs-3 (3 wt. part of CNTs/100 wt. parts of paint solids).

**Table 2:** Viscosity of the intumescent paints and results of the furnace test and thermogravimetric analysis of the intumescent coatings with CNTs or PER-CNTs solid disperions.

Sample Symbol	CNTs Dose ^1^	Paint Viscosity (Pa^.^s)	Thermal Insulation Time, TIT (Min)	Intumescent Factor, IF (a.u.)	Weight Loss Temperature ^2^ (°C)			Calcination Residue ^3^ (wt.%)
					T_5_	T_30_	T_50_	
R-0	0	0.59	25.4	9.9 ± 1.1	261	366	464	26.5
R-P1	1 ^4^	0.78	19.6	4.4 ± 1.1	262	375	477	25.4
R-P2	2 ^4^	0.97	12.9	1.3 ± 0.2	255	366	522	28.1
R-D1	1 ^5^	0.60	28.7	17.6 ± 2.7	266	373	482	26.9
R-D2	2 ^5^	0.59	30.0	17.9 ± 1.4	259	370	465	25.9
R-D3	3 ^5^	0.61	28.8	20.0 ± 1.0	249	361	466	27.1

^1^— wt. parts of the nanofiller/100 wt. parts of paint solids; ^2^—temperature at 5, 30 and 50% mass loss (thermogravimetric analysis of the coatings); ^3^—at 900 °C; ^4^—in the powder form; ^5^—in the form of powdered PER-CNTs solid dispersion.
